# A qualitative, multi-framework methodology for analysing health information technology–related patient safety incidents

**DOI:** 10.3389/fdgth.2026.1786358

**Published:** 2026-05-29

**Authors:** Md Shafiqur Rahman Jabin

**Affiliations:** 1 Centre for Digital Innovations in Health and Social Care, Faculty of Health and Social Care, University of Bradford, Bradford, United Kingdom; 2 eHealth Institute, Department of Medicine and Optometry, Linnaeus University, Kalmar, Sweden

**Keywords:** complex adaptive systems, HIT safety, incident reporting systems, patient safety learning, retrospective incident analysis, risk identification, safety governance, sociotechnical systems

## Abstract

**Background:**

The increasing reliance on health information technology (HIT) has introduced new and often unforeseen risks to patient safety in complex healthcare systems. Many HIT-related safety problems emerge only after systems are embedded in routine clinical practice and are difficult to identify using prospective or purely quantitative methods. Incident reports provide valuable insights into real-world failures, but systematic methodologies for analysing HIT-related incidents remain underdeveloped.

**Objective:**

This article aims to describe and formalise a qualitative, multi-framework methodology for analysing health information technology–related patient safety incidents, based on retrospective incident report data.

**Methods:**

The methodology integrates multiple data sources, including incident reporting systems, existing incident databases, and supplementary interview-derived narratives. HIT-related incidents are identified through a structured screening process combining keyword-based searches and manual narrative review. Analysis is conducted using complementary deductive and inductive approaches, including established patient safety classification systems, HIT-specific frameworks, workflow-based analysis, and thematic analysis. Structured coding procedures, independent review, consensus-building, and reflexive practices are employed to enhance analytical rigour. Findings are systematically translated into preventive and corrective strategies grounded in sociotechnical principles.

**Results:**

The proposed methodology enables systematic identification and characterisation of HIT-related patient safety incidents, capturing sociotechnical mechanisms, contributing factors, and outcomes that are not readily identified through single analytical frameworks. By combining multiple perspectives, the approach supports analysis of low-frequency, high-impact events, workflow disruptions, and system-level failures, and facilitates the development of context-sensitive preventive and corrective strategies.

**Conclusions:**

This multi-framework qualitative methodology provides a structured, transferable approach to learning from HIT-related patient safety incidents in complex healthcare systems. The framework supports researchers, clinicians, and safety analysts in understanding how digital systems fail in real-world practice and offers a robust foundation for improving the safety and resilience of digital healthcare.

## Introduction

Over the last few decades, healthcare systems have undergone substantial transformation driven by the rapid expansion of health information technology (HIT) (1). Digital systems now underpin many aspects of care delivery, including medical imaging (2–5), electronic prescribing (6), patient monitoring (7–9), and clinical documentation (9–11). These systems have been introduced to improve efficiency, accuracy, and patient safety. However, experience has shown that the expansion of HIT into routine clinical practice has not been seamless. When HIT systems are poorly planned, designed, implemented, integrated, or managed, they can introduce new risks to healthcare quality and patient safety, often in unforeseen and difficult-to-anticipate ways (1).

Healthcare delivery operates as a complex sociotechnical system in which interactions between humans, technologies, and organisational processes shape both performance and risk (12, 13). Within such systems, safety problems rarely arise from a single failure or isolated technical fault (1). Instead, incidents emerge from interactions among human actions, system design characteristics, workflow pressures, and latent organisational conditions (7, 14). These interactions can lead to a wide range of consequences, from inconvenience and workflow disruption (2, 7) to serious patient harm (1) and large-scale system failures that affect multiple patients or services simultaneously (1, 11).

The increasing reliance on HIT has added further layers of complexity to healthcare systems. Digital technologies can alter established work practices, redistribute cognitive workload, and create new dependencies between system components (15). While many of these changes bring benefits, they may also obscure failure mechanisms and make it more difficult for frontline staff to recognise, predict, or recover from system problems (1, 16). As a result, some of the most significant risks associated with HIT are not readily identifiable through prospective evaluation or controlled testing but become apparent only after systems are embedded in routine clinical use.

Understanding how and why things go wrong in such environments requires methods capable of capturing real-world complexity (1, 17). Prospective study designs and quantitative performance measures are valuable for assessing predefined outcomes, but they are limited in their ability to identify low-frequency, high-impact events and the contextual factors that shape them. In contrast, incident reports, i.e., free-text narratives generated by healthcare professionals after something has gone wrong, provide a unique source of information about failures as they occur in everyday practice (17–19). These reports allow insight into the circumstances surrounding incidents, the contributing human and technical factors, and the outcomes for patients, staff, and organisations (1, 20).

Incident reporting systems were not originally designed as research tools, and concerns have been raised regarding under-reporting, variability in report quality, and potential bias (1, 21). Nevertheless, within patient safety research, retrospective analysis of incident reports is recognised as one of the few practical methods for identifying rare but serious events in complex systems (1, 22). Most healthcare incidents occur infrequently and unpredictably, making systematic prospective observation impractical. Capturing information after events have occurred, therefore, remains essential for identifying patterns of failure, characterising risks, and informing preventive and corrective strategies (1, 22).

While incident reports provide valuable insights into real-world failures, they represent only one component of the broader safety learning ecosystem within healthcare organisations. Other sources of safety intelligence, including routine clinical debriefings, patient complaints, direct observations, and structured chart reviews, may capture different dimensions of safety-related issues. Recent work has demonstrated that these sources can provide complementary perspectives; for example, routine clinical debriefings tend to highlight issues related to teamwork, internal organisation, and procedural aspects of care, whereas incident reports more often capture problems related to care processes, patient flow, and patient-related concerns (23). Recognising these complementary sources is important for situating incident report analysis within a wider system of organisational learning. The methodology presented in this article is designed to support in-depth analysis of incident report data, while remaining compatible with the integration of additional data sources where available.

A further challenge in analysing HIT-related incidents lies in the limitations of any single analytical framework. Classification systems vary in their focus and scope; some emphasise clinical processes and outcomes, while others focus on technical failures or human–computer interaction (22, 24, 25). Experience across multiple studies has shown that no single framework is sufficient to characterise the breadth of issues arising from HIT in healthcare (1). Meaningful analysis requires complementary approaches that enable incidents to be examined from multiple perspectives, including contributing factors, system mechanisms, workflow stages, and emergent themes (22, 26).

A series of prior studies has examined HIT-related incidents across multiple clinical contexts, consistently demonstrating that such problems arise from complex interactions between human and technical factors and can be analysed to inform system-level improvements (1, 6, 8). These studies examined incidents related to medical imaging (2–4), e-prescribing (6), patient details (9), system configuration and upgrades (27), software patching (7), and large-scale events affecting multiple patients (11). Consistently, they demonstrated that HIT-related problems arise from complex interactions between human and technical factors and that human-related issues frequently lead to more deleterious outcomes than technical failures alone. Importantly, these studies also showed that systematic qualitative analysis of incident reports can be used not only to characterise problems but also to develop practical, sociotechnically informed strategies for improving safety and the quality of care.

The purpose of this article is to present a structured, qualitative, multi-framework methodology for analysing HIT-related patient safety incidents. The framework integrates retrospective incident data, deductive classification systems, workflow-based analysis, and inductive thematic approaches to support systematic and transferable analysis of complex sociotechnical risks. While elements of this approach have been applied in prior studies, this article brings these components together into a coherent and explicitly articulated methodological framework for analysing HIT-related patient safety incidents. The contribution is methodological rather than empirical, providing a transferable framework for systematic analysis across digital healthcare settings. The framework is explicitly grounded in a sociotechnical perspective and is intended to address the limitations of single-method approaches by enabling multi-dimensional analysis of complex, real-world HIT-related safety events.

The proposed methodology is derived from a programme of empirical research, including both multi-incident studies and in-depth case analyses conducted across diverse clinical contexts. While these studies applied elements of the approach in specific settings, the present article consolidates, abstracts, and formalises these elements into a coherent and transferable methodological framework. Table 1 summarises the key underpinning studies, their analytical approaches, and their specific contributions to the development of the framework.

**Table 1:** Empirical studies underpinning and informing the development of the proposed methodological framework.

Study	Study type	Data source/context	Analytical approaches applied	Key methodological contribution to the framework
Jabin (1–5)	Doctoral research/Conference Publications	Medical imaging incident reports	ICPS classification; qualitative analysis	Established a core approach to incident identification, classification, and sociotechnical interpretation
Jabin & Hammar (6)	Empirical study	E-prescribing incident reports	Deductive classification + thematic analysis	Demonstrated need for combining deductive and inductive approaches
Jabin et al. (11)	Empirical study	Multi-patient HIT incidents	Thematic + system-level analysis	Introduced analysis of large-scale/system-level events
Jabin et al. (10)	Empirical study	Software-related incidents	Thematic clustering	Identified recurring system-related patterns and failure clusters
Jabin et al. (9)	Empirical study	Patient details incidents	Classification + thematic analysis	Highlighted data integrity and patient identification issues
Pan et al. (8)	Empirical review	National incident datasets	System-focused analysis	Strengthened cross-context applicability and generalisability
Jabin (7, 27, 28)	Case studies	Software patching/System configuration/Loss of Central Surveillance Data	In-depth qualitative analysis	Demonstrated importance of configuration, updates, and operational disruption
Jabin et al. (2–5)	Empirical studies	Medical imaging workflow incidents	Workflow modelling + classification	Contributed to the development of a workflow-based analytical component

## Methodological epistemology and research design

**Figure 1:**
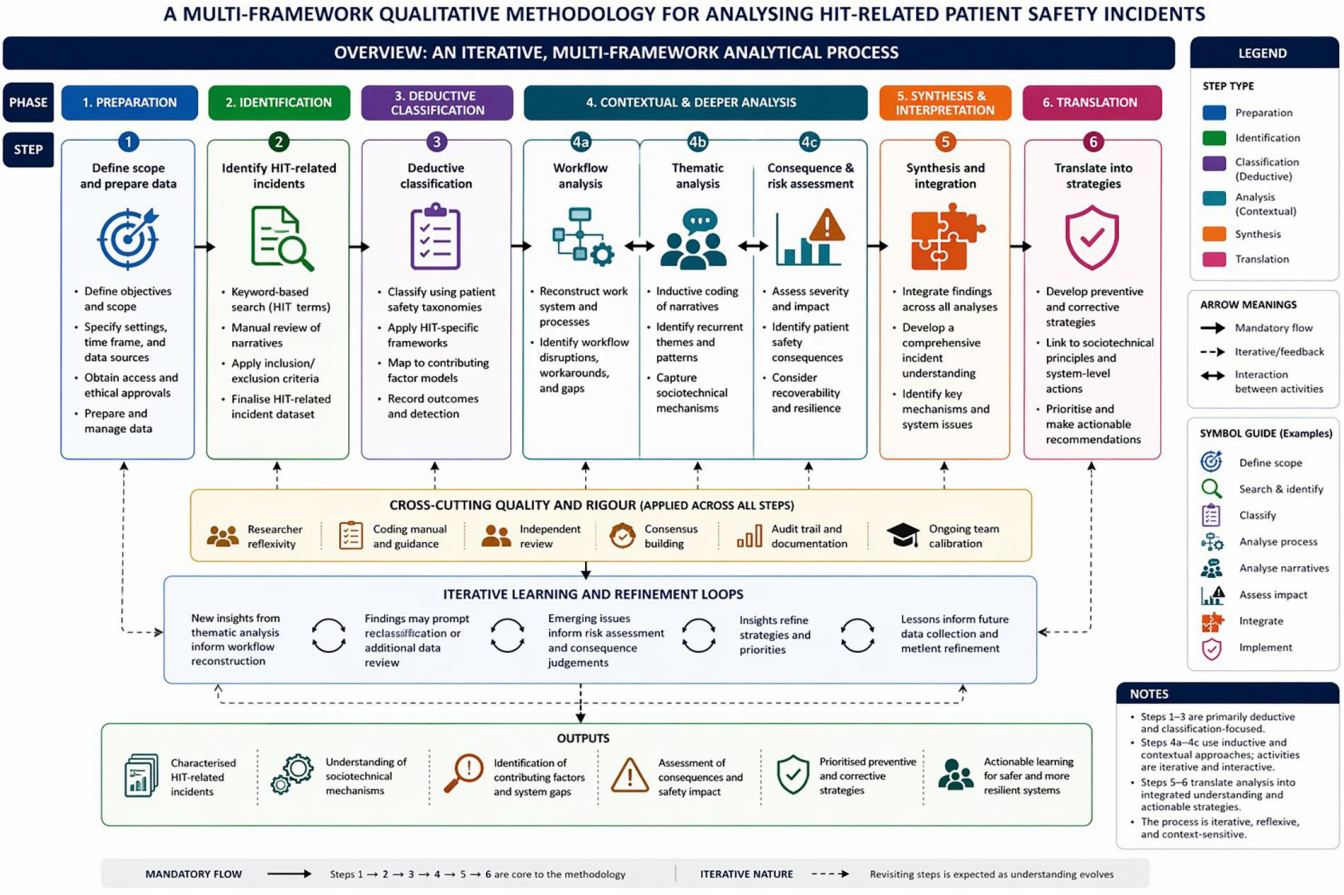
Operational workflow of the multi-framework methodology for analysing HIT-related patient safety incidents. The process comprises six core stages: data preparation and incident identification; deductive classification; multi-layered analysis; synthesis; and translation into strategies. While the overall workflow follows a structured sequence, several stages (particularly analytical and interpretive phases) are iterative and interact dynamically. Cross-cutting practices such as reflexivity, consensus-building, and audit trails are applied throughout to support methodological rigour and reproducibility.

The methodological approach adopted in this work is grounded in the epistemology of patient safety research, which conceptualises healthcare as a complex sociotechnical system in which adverse events emerge from interactions between human actors, technologies, workflows, and organisational conditions. Within such systems, safety problems rarely arise from a single failure or isolated technical fault; instead, they evolve through combinations of human actions, system design characteristics, and latent organisational factors. This perspective underpins much of the patient safety literature and has been central to the methodological orientation adopted throughout this body of work (1, 18). The current work differs from prior studies in that it focuses on formalising the methodological approach itself rather than reporting new empirical findings. An overview of the qualitative, multi-framework methodology used to identify, analyse, and interpret HIT-related patient safety incidents is shown in Figure 1.

Across the studies underpinning this methodology, healthcare incidents, particularly those involving HIT, i.e., were treated not as discrete errors but as manifestations of broader system vulnerabilities. Findings from analyses of medical imaging, e-prescribing, patient details, and system-level incidents consistently demonstrated that problems associated with HIT often arise only after systems are embedded in routine clinical practice, where real-world complexity, workflow pressures, and human–technology interactions become fully apparent (1, 6, 8). This reinforces the view that prospective testing and controlled evaluations, while necessary, are insufficient on their own to identify many clinically significant risks associated with digital healthcare systems.

Within this epistemological framework, retrospective analysis of incident reports is considered an essential method for learning from failure in complex systems. Incident reports, typically recorded as free-text narratives by healthcare professionals after events have occurred, provide insight into the circumstances surrounding incidents, perceived contributing factors, and observed outcomes. Although such reports were not originally designed for research purposes and are subject to limitations such as under-reporting and variability in narrative quality, they remain one of the few practical data sources capable of capturing low-frequency, high-impact events that are otherwise difficult to study systematically (1, 11, 29).

The research design underlying this methodology adopts a qualitative, retrospective, multi-stage approach to incident analysis. This design reflects experience gained across multiple empirical studies, which demonstrated that no single analytical framework is sufficient to characterise the breadth and complexity of HIT-related incidents. Early analyses using the International Classification for Patient Safety (ICPS) highlighted the value of a standardised framework for describing incident types, contributing factors, outcomes, and severity of harm (1, 22). However, subsequent work showed that the ICPS alone does not adequately capture sociotechnical mechanisms specific to HIT, such as software behaviour, system configuration, interoperability failures, or human–computer interaction issues (1, 6).

To address these limitations, the research design integrates multiple complementary analytical approaches. Classification systems tailored to HIT are used to characterise technical and use-related issues and to distinguish between human and technical contributing factors (1, 11). Workflow-based analysis is employed to situate incidents within the temporal structure of clinical processes, enabling examination of when problems arise, how they propagate across system boundaries, and where opportunities for detection or recovery may exist (1). This approach has been particularly valuable in identifying points in clinical workflows where HIT-related problems disproportionately affect patient safety, such as during referral, data entry, system configuration, or information transfer.

In addition to deductive classification, inductive thematic analysis is incorporated to explore aspects of incidents that are not well captured by existing frameworks. Thematic analyses conducted across multiple studies identified recurring clusters of problems, including issues related to patient details, incidents affecting the care of multiple patients, and system-level failures associated with upgrades, patching, or configuration changes (1, 7, 11, 22, 27). These findings demonstrated that inductive methods are essential for revealing emergent risk patterns and for refining analytical frameworks in response to evolving digital healthcare practices.

Overall, the methodological design reflects a pragmatic and iterative approach to studying patient safety in digital healthcare systems. It acknowledges the inherent limitations of retrospective incident data while recognising their unique value for identifying rare but consequential events and for understanding how sociotechnical systems fail in practice. By combining multiple analytical frameworks with qualitative methods, the approach supports a more comprehensive characterisation of HIT-related risks and provides a structured basis for developing preventive and corrective strategies grounded in empirical evidence from real-world clinical environments rather than theoretical assumptions alone (1, 11, 22).

## Data sources and sampling strategy

The data sources and sampling strategies employed in this methodological framework were selected to support the systematic study of HIT–related patient safety incidents in complex healthcare systems. Given the focus on understanding how and why things go wrong in routine clinical practice, the methodology prioritises real-world incident data generated in everyday healthcare delivery over data collected under controlled or experimental conditions. This approach reflects the recognition that many clinically significant HIT-related risks are low-frequency, context-dependent, and emergent, and therefore unlikely to be captured through prospective observation alone (29, 30).

### Data sources

The primary data source across the studies underpinning this methodology consists of retrospectively collected incident reports. These reports are typically recorded as free-text narratives by healthcare professionals following an incident, near miss, or hazardous situation. Incident reports were obtained from multiple sources, including local and regional healthcare incident reporting systems, national reporting infrastructures, and, in some cases, existing incident databases maintained by healthcare organisations. The use of multiple sources reflects both practical considerations, such as variability in access to reporting systems and methodological intent, allowing examination of incidents across different organisational contexts and clinical domains (1, 9, 11, 21).

In addition to incident reporting systems, supplementary data were collected through interviews and written responses from healthcare professionals, including clinicians, medical engineers, and healthcare quality managers. These data were used either to contextualise reported incidents or to capture incidents that had not been formally documented in reporting systems. Interview-derived incident narratives were treated analytically in the same manner as written incident reports, recognising that both represent retrospective accounts of events shaped by the reporter's perspective and local context (1, 11). This combination of data sources supports triangulation and helps mitigate some of the limitations of relying on a single reporting mechanism. Inclusion and exclusion decisions were made by the primary researcher and, where uncertainty arose, were reviewed in discussion with a second researcher to support consistency.

### Inclusion and exclusion criteria

Incident reports were eligible for inclusion if they described events in which HIT played a role in the occurrence, detection, or propagation of a patient safety issue. HIT was broadly defined to include digital systems used for clinical documentation, medical imaging, prescribing, decision support, communication, and information management (31). Incidents involving both direct and indirect interactions with HIT were considered eligible, recognising that digital systems often influence care processes in less visible ways, such as through data availability, system configuration, or workflow integration (1).

Reports were excluded if they lacked sufficient narrative detail to support meaningful qualitative analysis or if they could not reasonably be interpreted as involving HIT. Exclusion decisions were made conservatively, with ambiguous cases retained for further review where possible. This approach reflects the understanding that overly restrictive inclusion criteria risk excluding incidents that may reveal important sociotechnical mechanisms (32).

### Sampling strategies

Given the exploratory and explanatory aims of the methodology, probabilistic sampling was neither feasible nor appropriate. Instead, purposive sampling was employed to identify incidents and participants most likely to provide relevant information about HIT-related risks. This included targeting healthcare settings, professional roles, and clinical domains known to rely heavily on digital systems, such as medical imaging and electronic prescribing (1, 6).

In several studies, purposive sampling was complemented by snowball sampling, particularly when access to incident data or knowledgeable participants was limited. Initial contacts were asked to identify colleagues or departments with experience of HIT-related incidents, thereby extending the sample in a targeted manner. While snowball sampling may introduce selection bias, it is well-suited to studying specialised or under-recognised phenomena, such as rare HIT-related failures, where relevant expertise is unevenly distributed across organisations (33).

The goal of sampling was not statistical representativeness but analytical sufficiency. Sampling continued until a diverse range of incident types, contributing factors, and outcomes had been identified and until further data collection was unlikely to yield substantially new insights. This concept of information power aligns with qualitative research principles and supports the development of rich, conceptually meaningful analyses rather than frequency-based generalisations (34).

### Scope and transferability

Across the studies informing this methodology, incident reports were collected from multiple clinical domains and organisational settings over extended time periods. This breadth allowed examination of both recurring patterns and context-specific issues, as well as identification of large-scale events affecting multiple patients or services. Although the data are contextually situated, the methodological emphasis on mechanisms rather than event counts supports the transferability of findings across settings with similar sociotechnical characteristics (1, 11).

The sampling strategy is therefore best understood as supporting methodological generalisation rather than statistical inference. By focusing on how HIT-related incidents arise, propagate, and cause harm or disruption, the approach provides insights applicable to a wide range of digital healthcare systems, even when specific technologies or organisational contexts differ.

## Identification and selection of Hit-related incidents

The identification and selection of HIT–related incidents represent a critical methodological stage, as digital technologies are often embedded within broader clinical and organisational processes and may not be explicitly recognised as contributors to patient safety events. The approach adopted in this methodology reflects the understanding that HIT-related problems frequently manifest indirectly, through workflow disruption, information errors, or communication breakdowns, rather than as overt system failures (1, 11).

### Initial identification of incident reports

Incident identification begins with collecting retrospective incident reports from the data sources described in the previous section. These reports are typically recorded as free-text narratives and may vary considerably in length, structure, and level of technical detail. At this stage, all incident reports within the defined sampling frame are retained for screening, regardless of whether HIT involvement is explicitly stated. This inclusive approach is adopted to avoid prematurely excluding incidents in which digital systems play a contributory but less visible role.

### Screening for HIT relevance

Identification of HIT-related incidents is conducted through a structured screening process combining keyword-based searches with manual narrative review. Keyword lists are developed iteratively, informed by prior studies and refined through pilot screening. Keywords typically relate to digital systems, software, hardware, interfaces, data, documentation, system access, configuration, upgrades, and interoperability. However, keyword screening alone is recognised as insufficient, as many incident narratives describe HIT-related problems without using technical terminology (1, 35).

Consequently, keyword screening is followed by manual review of incident narratives to determine whether HIT contributed to the incident's occurrence, detection, or consequences. HIT involvement is defined broadly to include direct system failures, human–technology interaction issues, workflow disruptions mediated by digital systems, and latent system conditions such as configuration errors or data integrity problems (1, 36, 37).

### Inclusion criteria for HIT-related incidents

Incidents are classified as HIT-related if they meet one or more of the following criteria (1, 13, 36–39):

- A digital system directly malfunctioned or behaved in an unintended manner.
- A human interaction with a digital system contributed to the incident.
- Information generated, stored, or transmitted by a digital system was incorrect, missing, delayed, or misinterpreted.
- The incident involved system integration, configuration, upgrade, or interoperability issues.
- The digital system influenced clinical decision-making, workflow, or communication in a way that contributed to patient safety risk.

This inclusive definition reflects the sociotechnical perspective underpinning the methodology and acknowledges that HIT-related risks often arise from interactions rather than from isolated technical faults (1, 36–39).

### Exclusion criteria

Incidents are excluded if they clearly involve no interaction with digital systems or if the narrative lacks sufficient detail to assess HIT involvement despite follow-up clarification where feasible. Reports that mention digital systems only tangentially, without any plausible link to the incident's cause or outcome, are also excluded. Exclusion decisions are made conservatively to minimise the risk of omitting relevant sociotechnical failures (30, 40).

### Validation and consistency checks

To enhance methodological rigour, a subset of incident classifications is independently reviewed by a second researcher with expertise in patient safety and health informatics. Discrepancies regarding HIT relevance are discussed and resolved through consensus. This iterative review process supports consistency in incident identification and reduces the influence of individual interpretation, particularly in cases where HIT involvement is indirect or contested (24, 41). Screening decisions were primarily conducted by one researcher, with ambiguous cases reviewed in consultation with a second researcher to support consistency and minimise misclassification.

### Output of the identification stage

The outcome of this stage is a curated subset of incident reports identified as involving HIT, which then form the basis for subsequent deductive and inductive analyses. Importantly, this subset is not treated as exhaustive or representative of all HIT-related incidents within a system; rather, it provides analytically rich cases for systematically examining sociotechnical mechanisms, contributing factors, and outcomes (18, 20). Table 2 summarises the relationship between data sources and the analytical stages applied in the subsequent phases of the methodology.

**Table 2:** Mapping of data sources to analytical stages within the methodological framework for identifying and analysing HIT–related patient safety incidents.

Data source	Purpose	Analytical stage(s)	Methods applied	Outputs
Incident reporting systems (local, regional, national)	Primary source of real-world safety events	Identification; classification; analysis	Narrative screening; ICPS; HIT-CS; workflow mapping; thematic analysis	HIT-related incident subset; classified incidents; emergent themes
Existing incident databases	Longitudinal and large-scale event identification	Identification; comparative analysis	Retrospective filtering; framework-based coding	Patterns of recurring and large-scale HIT incidents
Interviews (written and telephone)	Supplementary incident capture and contextualisation	Identification; interpretation	Narrative analysis; triangulation with reports	Enhanced contextual understanding of incidents
Keyword lists and screening algorithms	Initial filtering for HIT relevance	Identification	Iterative keyword screening; manual review	Candidate HIT-related incidents
Deductive classification frameworks (ICPS, HIT-CS)	Structured characterisation of incidents	Classification; comparison	Framework-based coding	Incident types, contributing factors, and outcomes
Workflow models	Temporal and process-based localisation of failures	Analysis	Mapping incidents to workflow stages	Identification of high-risk workflow points
Inductive thematic analysis	Identification of emergent risk patterns	Analysis; synthesis	Thematic coding and clustering	Cross-cutting themes (e.g., patient details, system issues)
Researcher consensus review	Validation and consistency	Identification; classification	Independent review and discussion	Improved reliability and methodological transparency

## Analytical frameworks

Following the identification and selection of HIT–related incidents, the subsequent analytical phase applies a set of complementary frameworks to characterise incidents from multiple perspectives. Table 3 provides an overview of the analytical frameworks applied and their respective purposes within the methodology. As summarised in Table 1, different data sources inform different analytical stages, reflecting the recognition that no single framework is sufficient to capture the complexity of HIT-related patient safety incidents. Instead, the methodology adopts a layered analytical approach that combines deductive classification systems with inductive qualitative analysis and workflow-based perspectives.

**Table 3:** Analytical frameworks applied and their analytical purpose.

Analytical approach	Framework/method	Analytical focus	Output
Deductive classification	ICPS	Incident types, contributing factors, and outcomes	Structured incident characterisation
HIT-specific classification	HIT safety frameworks	Sociotechnical mechanisms	Human vs. technical contributors
Workflow analysis	Process/workflow models	Temporal localisation of failures	High-risk workflow stages
Inductive analysis	Thematic analysis	Emergent patterns and clusters	Cross-cutting themes

This multi-framework approach is grounded in sociotechnical theory and patient safety research, which emphasise that incidents in complex systems arise from interactions between human, technical, and organisational elements rather than from isolated failures (12, 42). Applying multiple analytical lenses allows these interactions to be examined systematically and supports both structured comparison across incidents and the identification of emergent risk patterns.

### Deductive analytical frameworks

Deductive analysis is used to provide structure and consistency in the characterisation of incidents and to support comparison across datasets, clinical domains, and organisational contexts. Two types of deductive frameworks are employed: general patient safety classifications and HIT-specific classification systems (29, 38, 43–45).

The International Classification for Patient Safety (ICPS) is used to classify incident types, contributing factors, outcomes, and degree of harm using a shared conceptual framework and standardised terminology (24). The ICPS enables the systematic description of what happened, what contributed to the incident, and the resulting outcomes, facilitating comparisons across incidents and supporting aggregation at different levels of analysis. Its use reflects the importance of a common patient safety language when analysing incidents reported by diverse professional groups and organisations (30, 46).

However, general patient safety classifications alone are insufficient to capture the sociotechnical mechanisms specific to digital systems. To address this limitation, HIT-specific classification frameworks are applied to identify and characterise issues related to software behaviour, hardware performance, system configuration, interoperability, and human–computer interaction. Such frameworks have been shown to be essential for distinguishing between human-related and technical contributing factors and for understanding how digital systems shape clinical work practices (29, 37, 38).

Deductive coding using these frameworks is not treated as a purely mechanical process. Instead, incident narratives are interpreted in context, recognising that a single incident may be associated with multiple contributing factors and may span more than one classification category. This approach aligns with prior work demonstrating that rigid single-category assignment risks oversimplifying complex sociotechnical events (47).

### Workflow-based analysis

In addition to classification-based approaches, workflow-oriented analysis is used to situate incidents within the temporal and operational structure of clinical processes. Workflow models provide a means of examining when incidents occur, how they propagate across system boundaries, and where opportunities for detection, recovery, or mitigation may exist. This perspective acknowledges that the impact of HIT-related problems often depends not only on the nature of the failure but also on its timing and position within a broader care pathway (2, 48).

Mapping incidents to workflow stages supports identifying process points that are particularly vulnerable to HIT-related disruptions, such as data entry, information transfer, system access, or result communication. Workflow-based analysis also facilitates examination of interactions between digital systems and human roles at different stages of care, consistent with sociotechnical models of work system design (2, 12, 13).

### Inductive thematic analysis

While deductive frameworks provide structure, they do not capture all aspects of complex incidents, particularly those that fall outside predefined categories or reveal emerging risk patterns. To address this, inductive thematic analysis is employed to explore incident narratives without imposing *a priori* classifications. This approach enables the identification of recurring themes, clusters of related problems, and cross-cutting issues that may not be well represented within existing frameworks (49, 50).

Thematic analysis is conducted iteratively, with codes and themes refined as analysis progresses. Themes are developed from patterns observed across incidents, such as issues with patient details, system configuration changes, large-scale events affecting multiple patients, or workarounds adopted by staff in response to system limitations. The inductive findings are then considered alongside deductive classifications, allowing convergence and divergence between analytical approaches to be examined.

### Integration of analytical approaches

The final stage of analysis involves integrating findings from deductive classification, workflow mapping, and thematic analysis. Rather than privileging one framework over another, the methodology treats these approaches as complementary. Deductive frameworks support consistency and comparability, workflow analysis situates incidents within care processes, and thematic analysis reveals emergent sociotechnical patterns (1, 2, 51).

This integrative approach reflects the understanding that complex digital healthcare systems cannot be adequately analysed using a single perspective. By triangulating findings across multiple analytical frameworks, the methodology supports a more comprehensive characterisation of HIT-related risks and provides a robust foundation for developing preventive and corrective strategies grounded in real-world practice (15, 36, 51).

The methodology is implemented as a structured, stepwise process consisting of six core stages (Figure 1). Stages 1–3 (preparation, identification, and deductive classification) constitute the core sequential workflow for establishing a consistent analytical foundation. Stages 4–6 (analytical integration, consequence assessment, and translation) are applied iteratively, allowing findings from one stage to inform refinement in others. While all stages are recommended for comprehensive analysis, the methodology is designed to be flexible, enabling adaptation to data availability and research objectives.

## Coding procedures and reliability

The coding procedures applied in this methodological framework were designed to ensure systematic analysis of incident narratives while acknowledging the interpretive nature of qualitative patient safety research. Given that incident reports are free-text accounts produced retrospectively by healthcare professionals, coding is treated as an analytic process rather than a purely mechanical task. The procedures, therefore, combine the structured application of analytical frameworks with iterative interpretation and verification.

### Coding process

Coding is conducted in multiple stages corresponding to the analytical frameworks described in Section 5. Following identification of HIT-related incidents, each incident narrative is reviewed in full and coded deductively using predefined classification systems to characterise incident type, contributing factors, and outcomes. Where applicable, multiple codes may be assigned to a single incident to reflect the presence of more than one contributing factor or outcome, consistent with the understanding that patient safety incidents often involve interacting causes rather than linear chains of failure (29, 42).

In parallel, workflow-based coding maps incidents to stages of clinical processes. This involves identifying the point(s) in the workflow at which the incident originated, was detected, or had its primary impact. Coding at this stage supports temporal localisation of failures and facilitates examination of how HIT-related problems propagate across system boundaries (12, 48).

Inductive coding is subsequently applied to incident narratives to identify emergent themes not adequately captured by deductive frameworks. This process follows established principles of thematic analysis, involving familiarisation with the data, generation of initial codes, and iterative development and refinement of themes (49, 50). Inductive codes are developed directly from the data and refined through comparisons across incidents, enabling the identification of recurring patterns and clusters of issues.

### Coding roles and verification

To enhance analytical rigour, coding is performed by at least one primary coder with expertise in patient safety and health information technology. A second coder independently reviews a subset of coded incidents to verify coding decisions and assess consistency. The subset selected for independent review typically includes incidents spanning multiple clinical domains, incident types, and severity levels to ensure broad coverage.

Discrepancies between coders are examined through discussion, and consensus is reached by re-examining the incident narrative and relevant coding guidelines. This consensus-based approach recognises that some degree of interpretive variation is inevitable when analysing complex sociotechnical events and prioritises shared understanding over strict numerical agreement (30, 52).

Coding was primarily conducted by a single experienced researcher with expertise in patient safety and health information technology. To enhance methodological rigour, a subset of incidents (typically 15%–25%, depending on dataset size and study context) was independently reviewed or double-coded by a second researcher. This subset was purposively selected to include a range of incident types, severity levels, and clinical contexts.

Interrater agreement was assessed for selected deductive coding dimensions, particularly those related to incident type, contributing factors, and outcomes, for which classification categories were well-defined. These procedures reflect practices applied across the empirical studies underpinning this methodology rather than being tied to a single dataset. Discrepancies were resolved through discussion and consensus, and coding frameworks were iteratively refined where necessary.

### Assessment of coding reliability

Where deductive classification frameworks are applied, interrater reliability is assessed using appropriate statistical measures, such as Cohen's kappa, to quantify agreement beyond chance. Reliability assessment is conducted for selected coding dimensions, particularly those related to contributing factors and outcomes, where classification categories are well defined (53). Reporting reliability metrics provides transparency regarding coding consistency and supports the credibility of the analytical process.

For inductive thematic analysis, reliability is addressed through methodological transparency, iterative refinement of codes, and reflexive discussion rather than through formal statistical measures. This approach aligns with qualitative research guidance emphasising trustworthiness, reflexivity, and analytic coherence over numerical indicators alone (34, 50).

### Reflexivity and analytic transparency

Throughout the coding process, reflexivity is maintained to acknowledge how researchers’ backgrounds, disciplinary perspectives, and prior experience may shape the interpretation of incident narratives. Analytic decisions, including code definitions, inclusion of ambiguous cases, and resolution of disagreements, are documented to support transparency and reproducibility (34, 50).

The combination of structured coding procedures, independent review, consensus-building, and reflexive practice supports a balanced approach to reliability that is appropriate for qualitative analysis of complex patient safety incidents. Rather than seeking to eliminate interpretation, the methodology aims to make interpretive processes explicit and systematically applied, thereby enhancing the credibility and usefulness of the findings for understanding HIT-related risks in real-world healthcare settings (29, 52).

## Assessment of consequences and impact

Assessment of consequences and impact is integral to the analytical process, as understanding the effects of HIT–related incidents is essential for interpreting their significance and informing improvement efforts. In this methodology, consequences are examined not only in terms of patient harm but also in terms of their impacts on healthcare professionals, workflows, and organisations. This broader perspective reflects the recognition that incidents in complex sociotechnical systems often produce multiple, interconnected outcomes that extend beyond immediate clinical effects (18, 42, 46).

### Conceptualisation of outcomes

Outcomes are conceptualised as the observable effects that are wholly or partially attributable to an incident. These may include patient-related outcomes, such as inconvenience, delayed care, or physical harm, as well as staff- and organisation-related outcomes, such as increased workload, workflow disruption, resource utilisation, or loss of trust in digital systems. This inclusive conceptualisation aligns with established patient safety frameworks, which emphasise that harm and impact can occur at multiple levels of the healthcare system (24, 29, 30, 46).

Importantly, the absence of documented patient harm does not imply the absence of risk. Near misses and incidents resulting primarily in organisational or workflow consequences are treated as analytically significant, as they may indicate latent system vulnerabilities with the potential to cause future harm under different circumstances (18, 42) (Reason, 2000; Hollnagel et al., 2015).

### Classification of consequences

Consequences are classified using structured patient safety frameworks to support consistency and comparability across incidents. Where applicable, established outcome categories are used to distinguish between patient-related outcomes and organisational outcomes, including service disruption and staff impact. Applying predefined categories facilitates aggregation and comparison while maintaining transparency regarding how outcomes are defined and interpreted (24, 29).

At the same time, outcome classification is informed by narrative context. Incident reports often vary in the level of detail provided regarding consequences, and outcomes may be implicit rather than explicitly stated. In such cases, cautious interpretation is applied, drawing only on information supported by the narrative and avoiding speculative attribution of harm. This conservative approach aligns with guidance in patient safety research on balancing sensitivity to potential harm against the limitations of retrospective reporting (30, 54, 55).

### Severity and scale of impact

In addition to categorising outcomes, the methodology considers the severity and scale of impact. Severity refers to the extent of harm or disruption resulting from an incident, ranging from no harm or minor inconvenience to severe harm or death. Scale refers to the number of patients, staff members, or services affected by an incident. This distinction is particularly important in the context of HIT, where system-level failures may affect multiple patients simultaneously or disrupt entire clinical services (1, 35, 38, 56).

Assessment of severity and scale supports identification of incidents with disproportionate impact and highlights the potential for digital systems to amplify errors across organisational boundaries. Even incidents resulting in limited immediate harm may be prioritised for analysis if they reveal failure modes capable of producing widespread consequences under slightly different conditions (18, 42, 57).

### Analytical role of consequence assessment

Assessment of consequences and impact is not treated as an endpoint of analysis but as an input to subsequent interpretation and strategy development. Understanding what was affected and to what extent helps contextualise contributing factors and informs judgments about the relative importance of the sociotechnical mechanisms identified in earlier analytical stages. In this way, consequence assessment contributes to a more holistic understanding of HIT-related incidents and supports the development of preventive and corrective strategies grounded in real-world risk rather than isolated event descriptions (20, 46).

## Translation of findings into preventive and corrective strategies

Translation of analytical findings into preventive and corrective strategies constitutes a central objective of this methodological framework. In patient safety research, the value of incident analysis lies not only in describing what went wrong, but in using empirical insights to inform interventions that reduce the likelihood or impact of future events. This methodology, therefore, treats strategy development as a systematic, evidence-informed process grounded in a sociotechnical understanding of HIT–related incidents arise in real-world clinical settings (1, 30, 55).

### Conceptual basis for strategy development

Preventive and corrective strategies are developed based on the recognition that incidents in complex healthcare systems emerge from interactions between human, technical, and organisational elements. As such, effective interventions rarely target a single component in isolation. Instead, strategies are designed to address system-level vulnerabilities identified through deductive classification, workflow analysis, and inductive thematic patterns. This approach aligns with sociotechnical and systems-based models of safety improvement, which emphasise redesign of work systems rather than reliance on individual vigilance or training alone (1, 13, 58).

Findings from incident analysis are first interpreted to identify underlying mechanisms of failure, such as information breakdowns, mismatches between system design and clinical workflow, or unintended consequences of system configuration and integration. These mechanisms provide the analytic link between observed incidents and potential points for intervention, supporting development of strategies that target root causes rather than surface symptoms (18, 43).

### Mapping findings to intervention domains

To support systematic translation, analytical findings are mapped to broad intervention domains, including system design, workflow and process redesign, training and support, governance and policy, and monitoring and feedback mechanisms. This mapping enables clustering of related strategies and helps ensure that interventions address both technical and human dimensions of HIT-related risks. For example, findings related to data integrity or interface design may inform system redesign or configuration changes, while findings related to workarounds or delayed documentation may indicate a need for workflow adaptation or organisational support (1, 35).

Importantly, the methodology avoids assuming a one-to-one correspondence between incident types and interventions. Multiple strategies may be appropriate for addressing a single mechanism of failure, and a single strategy may mitigate risks across several incident types. This flexibility reflects the complexity of digital healthcare systems and the need for layered defences rather than single-point solutions (1, 42).

### Preventive vs. corrective strategies

Preventive strategies aim to reduce the likelihood of future incidents by addressing latent system conditions before harm occurs. These may include changes to system design, improved interoperability, clearer information standards, or enhanced support during system implementation and upgrades. Corrective strategies, in contrast, focus on mitigating the consequences of incidents once they have occurred, such as improving detection mechanisms, strengthening recovery processes, or enhancing communication during system downtime or failure (1, 56, 59).

Both preventive and corrective strategies are considered essential components of safety improvement. Overemphasis on prevention alone may underestimate the inevitability of failure in complex systems, while reliance solely on corrective measures risks normalising unsafe conditions. The methodology, therefore, supports a balanced approach that combines anticipation, monitoring, response, and learning (60).

### Prioritisation and feasibility considerations

Strategy development also considers impact, feasibility, and context. Not all identified risks can be addressed simultaneously, and interventions vary in their resource requirements and organisational implications. Findings related to high-severity incidents or large-scale impacts are prioritised, particularly when they reveal vulnerabilities that could affect multiple patients or services. At the same time, strategies are assessed for practical feasibility, recognising constraints related to technology, staffing, regulation, and local context (30).

Rather than prescribing specific solutions, the methodology emphasises generating context-sensitive strategies that can be adapted to local conditions. This supports transferability across healthcare settings while acknowledging that implementation requires engagement with stakeholders, organisational leadership, and frontline staff (19, 20).

### Role of strategy development in the methodological framework

Within this framework, translating findings into preventive and corrective strategies constitutes a continuation of analysis rather than a separate phase. Strategy development is informed by all preceding analytical stages and may, in turn, highlight areas requiring further investigation or refinement of analytical frameworks. This iterative relationship underscores the role of incident analysis in a continuous learning cycle aimed at improving the safety and resilience of digital healthcare systems (1, 46, 61).

## Methodological strengths and limitations

Critical reflection on methodological strengths and limitations is essential for situating the findings derived from this framework and for supporting appropriate interpretation and use of results. The approach presented in this article is designed to balance rigour and flexibility to study complex, low-frequency, and sociotechnical patient safety incidents associated with HIT. As with all methodological approaches, it offers distinct strengths but is also subject to inherent limitations. Rather than representing a methodological weakness, this interpretive element is treated as an analytic resource and is managed through reflexivity, consensus-building, and transparent documentation.

### Methodological strengths

A key strength of this methodology lies in its grounding in real-world clinical practice. By drawing on retrospectively collected incident reports and narrative accounts from healthcare professionals, the approach captures events as they occur in routine care rather than under experimental or simulated conditions. This supports identification of emergent risks, unintended consequences, and system interactions that are difficult to anticipate prospectively, particularly in digitally mediated healthcare environments (30, 35, 38).

The use of multiple, complementary analytical frameworks represents another major strength. Combining deductive classification systems, workflow-based analysis, and inductive thematic analysis enables incidents to be examined from different perspectives and mitigates the limitations of relying on a single framework. This layered approach supports a more comprehensive characterisation of HIT-related incidents, allowing the analysis of contributing factors, mechanisms, and outcomes in relation to one another rather than in isolation (1, 12, 35).

Methodological transparency and rigour are further strengthened through structured coding procedures, independent review, consensus-building, and reflexive practice. Explicit documentation of analytic decisions and coding processes enhances credibility and supports reproducibility, particularly in the context of qualitative analysis of complex sociotechnical phenomena (1) Braun & Clarke, 2006). The inclusion of incidents across multiple clinical domains and organisational contexts also supports analytical generalisation by enabling comparison of patterns and mechanisms across settings (1, 51).

### Methodological limitations

Despite these strengths, several limitations must be acknowledged. Incident reporting systems are known to be affected by under-reporting, selective reporting, and variability in narrative quality. Not all incidents are reported, and those that are reported may over-represent certain types of events or perspectives, particularly those perceived as unusual or serious (54, 62, 63). As a result, the analysed incidents cannot be assumed to represent the full spectrum or frequency of HIT-related safety problems.

More recent evidence further highlights the extent to which voluntary incident reporting systems underestimate the true burden of patient harm. For example, a large retrospective review of inpatient admissions found adverse events in approximately 23.6% of cases, substantially exceeding rates typically identified through voluntary reporting systems (64). Similarly, previous studies have shown that certain categories of adverse events, such as adverse drug events, may be detected at significantly higher rates through systematic record review than through incident reporting alone. These findings reinforce the need to interpret incident report data as a partial and selective representation of safety events, shaped by reporting practices, organisational culture, and system design.

The retrospective nature of incident reports also limits the availability and precision of information. Narratives may lack detail regarding context, contributing factors, or outcomes, and causal relationships cannot be established with certainty. Interpretation, therefore, relies on the information provided by reporters and is subject to recall bias and subjective framing (30). While conservative inclusion criteria and validation procedures help mitigate these issues, they cannot eliminate them entirely.

Another limitation relates to the interpretive nature of qualitative analysis. Although structured frameworks and reliability checks are employed, analysis inevitably involves judgement. Different analysts may emphasise different aspects of an incident or interpret narratives differently. This limitation is addressed through reflexive practice, consensus-building, and transparent reporting, but it remains an inherent feature of qualitative sociotechnical research rather than a methodological flaw (52, 65).

Finally, while the methodology is designed to be transferable across contexts, findings derived from its application are influenced by local organisational, technological, and regulatory conditions. Strategies and insights generated through analysis, therefore, require contextual adaptation before implementation in other settings (20).

### Implications of strengths and limitations

Taken together, these strengths and limitations indicate that the methodology is best suited for exploratory and explanatory analysis of HIT-related patient safety incidents, rather than for estimating incidence rates or making statistical generalisations. Its value lies in revealing mechanisms, patterns, and vulnerabilities that can inform system design, policy, and quality improvement efforts. When interpreted within these boundaries, the approach provides a robust and credible means of learning from failure in complex digital healthcare systems (42, 46).

## Transferability and implications for research and practice

The methodological framework presented in this article is intended to be transferable across healthcare contexts in which digital systems play a central role in care delivery. Rather than being tied to a specific technology, organisation, or national reporting system, the approach focuses on underlying sociotechnical mechanisms through which HIT–related patient safety incidents arise. This emphasis on mechanisms rather than event frequency or local configurations supports application of the methodology across diverse clinical domains and healthcare settings. Future applications of the methodology may benefit from integrating incident report analysis with other sources of safety intelligence, such as debriefings or observational data, to provide a more comprehensive understanding of system performance and risk.

### Transferability of the methodology

The transferability of qualitative findings depends on conceptual relevance rather than on statistical representativeness. By grounding analysis in established patient safety and sociotechnical theories, and by making analytic processes explicit, the methodology enables readers to assess its applicability to their own contexts. The use of broadly applicable data sources, such as incident reporting systems and narrative accounts from healthcare professionals, further supports transferability, as such systems are widely used across healthcare organisations internationally (21, 40).

The framework is adaptable to different types of HIT, including electronic health records, e-prescribing systems, medical imaging platforms, and emerging digital health technologies. While specific system configurations and regulatory environments may vary, the core analytical stages, i.e., incident identification, multi-framework analysis, consequence assessment, and strategy development, remain applicable. Local adaptation is expected and encouraged, particularly with respect to workflow models, classification systems, and prioritisation of risks, which should reflect organisational structures and clinical practices (12, 13, 58).

### Implications for research

For researchers, the methodology provides a structured approach for studying low-frequency, high-impact HIT-related incidents that are difficult to capture through prospective designs. It offers a means of systematically analysing narrative incident data while maintaining sensitivity to context and complexity. The multi-framework approach also supports methodological triangulation, enabling researchers to combine standardised classification with exploratory qualitative analysis to generate richer insights than either approach alone (1, 31, 38, 49).

The framework may also inform the design of future studies by highlighting the value of incident reports as a primary data source for digital safety research. Researchers can apply the methodology longitudinally to examine how risks evolve over time, particularly in relation to system upgrades, configuration changes, or large-scale digital transformations. In addition, the approach can be combined with other qualitative or quantitative methods, such as observations, interviews, or system log analysis, to deepen understanding of identified mechanisms and support mixed-methods research designs (22, 52).

### Implications for clinical practice and safety management

For clinical practice and safety management, the methodology provides a structured way to learn from incidents beyond local case review. By systematically analysing incident narratives and identifying sociotechnical mechanisms, healthcare organisations can shift from reactive responses to more proactive, preventive safety strategies. The framework supports identification of vulnerabilities at the level of system design, workflow integration, and organisational processes, rather than focusing solely on individual errors (1, 18, 46).

The emphasis on translating findings into preventive and corrective strategies also aligns with contemporary approaches to safety management that prioritise learning and system resilience. Safety managers and digital health leaders can use the methodology to prioritise risks, inform governance decisions, and guide interventions related to system implementation, configuration, and training. Importantly, the framework encourages attention to incidents that may not result in immediate patient harm but signal latent risks with the potential for serious future consequences (1, 60).

### Policy and system-level implications

At a broader level, the methodology has implications for national and organisational incident reporting systems. Systematic application of such frameworks can enhance the analytical value of reported incidents and support aggregation of learning across organisations. By focusing on mechanisms and patterns rather than isolated events, the approach contributes to more meaningful use of incident data for policy development, regulation, and digital health governance (1, 46, 60).

As healthcare systems continue to undergo digital transformation, the need for robust methodologies to monitor, analyse, and learn from HIT-related risks will increase. The framework presented here offers a transferable, adaptable approach to meeting this need, complementing existing safety monitoring and quality improvement efforts and contributing to the development of safer, more resilient digital healthcare systems.

## Conclusion and methodological contribution

This article presents a qualitative, multi-framework methodology for analysing HIT–related patient safety incidents in complex healthcare systems. Responding to the limitations of prospective evaluations and single-method approaches, the methodology provides a structured yet flexible framework for learning from real-world incidents as they occur in routine clinical practice. This work builds on an established body of empirical research and contributes a consolidated methodological framework intended to support systematic and context-sensitive analysis of HIT-related patient safety incidents across diverse healthcare settings.

The methodological contribution of this work lies in its integration of complementary analytical approaches within a coherent framework. By combining systematic identification of HIT-related incidents, deductive classification using established patient safety frameworks, workflow-based analysis, inductive thematic analysis, and structured assessment of consequences, the methodology enables a comprehensive examination of the sociotechnical mechanisms underlying HIT-related safety problems. This layered approach supports analysis of low-frequency, high-impact events and system-level failures that are often difficult to capture using traditional study designs.

A further contribution is the explicit treatment of incident identification and coding as analytic acts requiring interpretive judgement, reflexivity, and transparency. By documenting coding procedures, validation processes, and reliability considerations, the methodology addresses common challenges associated with retrospective incident analysis and enhances credibility. While incident reports provide valuable insights into real-world failures, they represent only one component of the broader safety learning ecosystem within healthcare organisations. Other sources of safety intelligence, including routine clinical debriefings, patient complaints, direct observations, and structured chart reviews, may capture different dimensions of safety-related issues. Recent work has demonstrated that these sources can provide complementary perspectives; for example, routine clinical debriefings tend to highlight issues related to teamwork, internal organisation, and procedural aspects of care, whereas incident reports more often capture problems related to care processes, patient flow, and patient-related concerns (23). Recognising these complementary sources is important for situating incident report analysis within a wider system of organisational learning. The methodology presented in this article is designed to support in-depth analysis of incident report data, while remaining compatible with the integration of additional data sources where available.

Importantly, the framework is designed to support translation of analytical findings into preventive and corrective strategies grounded in system-level understanding rather than individual blame. This emphasis aligns with contemporary perspectives on patient safety and resilience and provides a practical bridge between incident analysis and safety improvement efforts in digital healthcare environments.

While the methodology does not aim to produce statistically generalisable results, it offers a transferable approach for understanding how digital systems contribute to patient safety risks across diverse clinical and organisational contexts. As healthcare systems continue to undergo rapid digital transformation, robust and transparent methodologies for analysing HIT-related incidents will be essential. The framework presented here contributes to this need by offering researchers, clinicians, and safety practitioners a systematic means of learning from failure and supporting the development of safer, more resilient digital healthcare systems.

## Data Availability

The original contributions presented in the study are included in the article/Supplementary Material, further inquiries can be directed to the corresponding author.
